# Supplementary material for: Nitric Oxide Mitigates the Deleterious Effects Caused by Infection of Pseudomonas syringae pv. syringae and Modulates the Carbon Assimilation Process in Sweet Cherry under Water Stress
Source: Plants (Basel). 2024 May 14;13(10):1361. doi: 10.3390/plants13101361 (PMC11125257; doi:10.3390/plants13101361)
Supplement: Supplementary file 1 [file plants-13-01361-s001.zip › plants-2975967-supplementary.pdf]

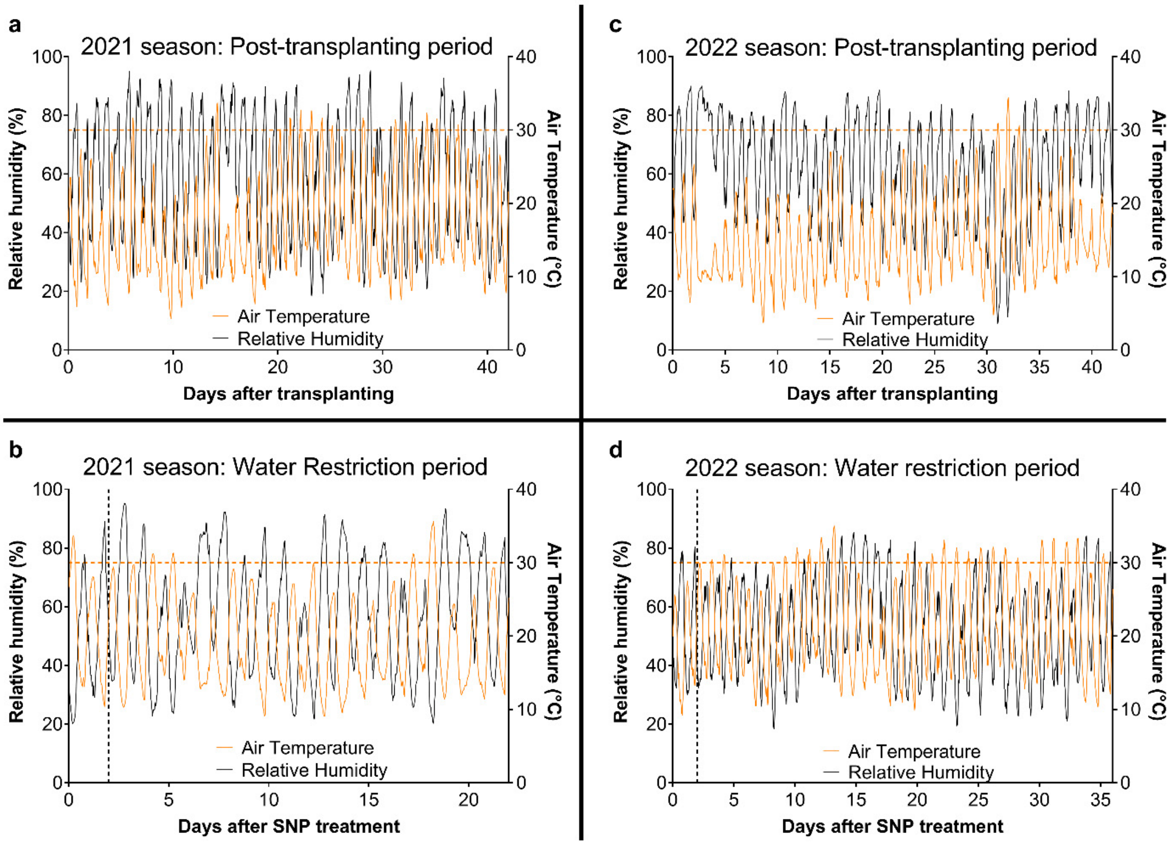

Supplementary Figure S1. Fluctuation of the air environmental parameters in the post-transplanting period and during the following water restriction assay. Air temperature (orange) and relative humidity (black) records in the post-transplanting period (a, b) and water restriction period (c, d) in the 2021 and 2022 seasons. The dashed black vertical line indicates the beginning of water withholding (two days after SNP application) over plants. The dashed orange horizontal line indicates the 30°C threshold.

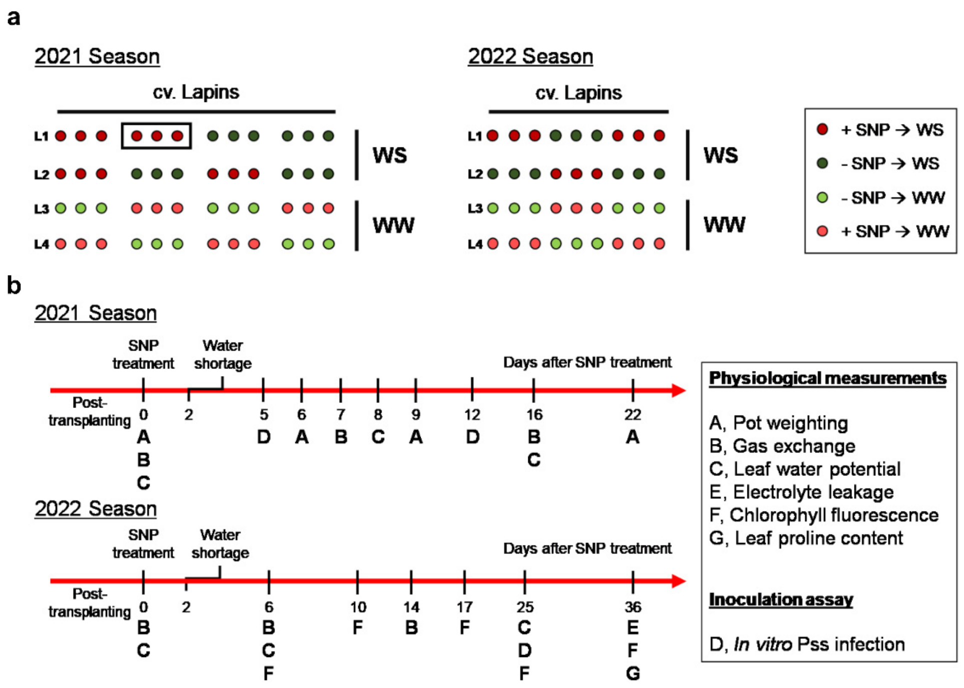

Supplementary Figure S2. Experimental design and measurements used for the water restriction assay. (a) Outline of the spatial disposition of plants of sweet cherry cv. Lapins during water restriction assay in the 2021 and 2022 seasons. Plants (indicated as circles) were treated with or without SNP 0.5 mM (+ SNP or – SNP, respectively). Irrigation was conducted using irrigation lines (L): L1 and L2, unlike L3 and L4 (WW), were cut two days after SNP treatment (WS). Three plants represented an experimental unit (highlighted with a box). The black arrow indicates a temporal water condition, WW or WS. **(b)** Temporal course of the main events of the water restriction assays performed in both seasons. First, there was a post-transplanting period (six weeks). After that, SNP treatment was performed and two days after, water shortage was applied. Six physiological measurements in addition to the *in vitro* Pss infection were performed. Each measurement is indicated by a letter, and the performing day is shown above its letter.

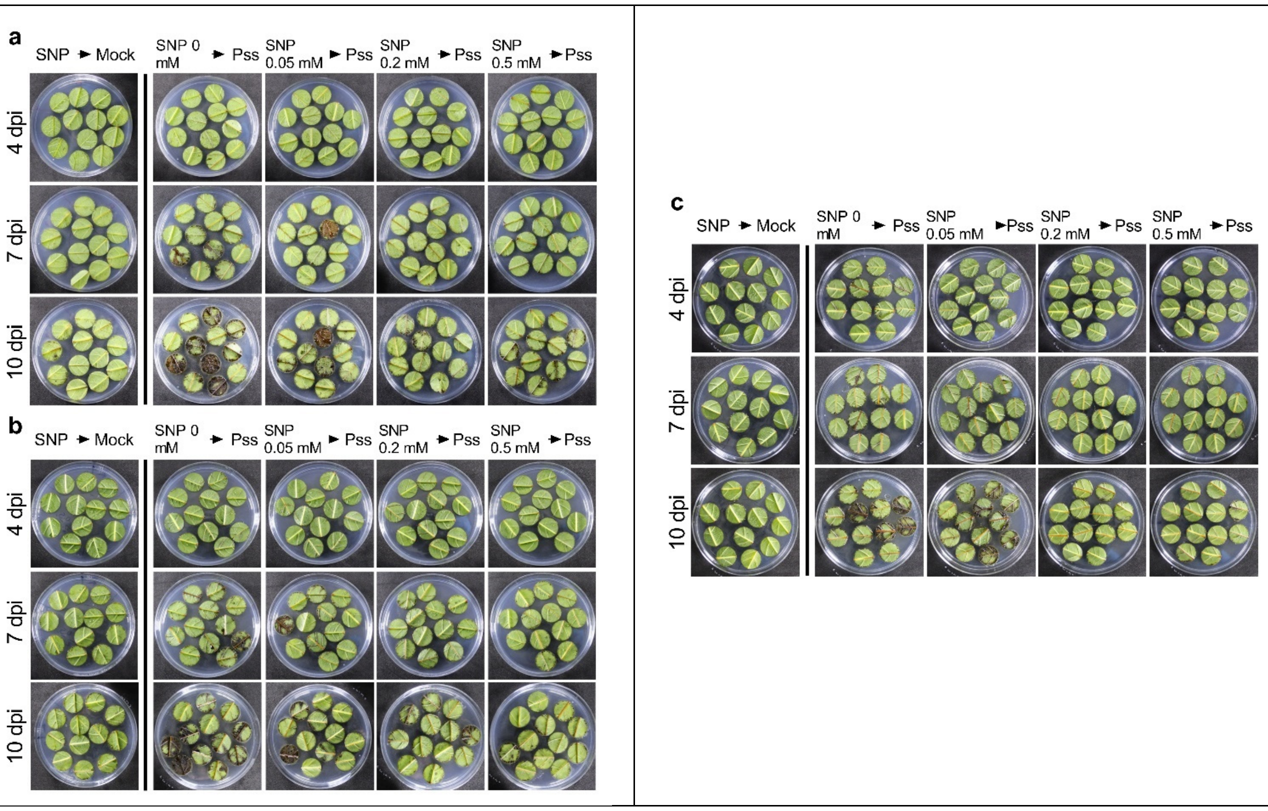

Supplementary Figure S3. ***In vitro*** **Pss infection in sweet cherry leaves treated with SNP.** Sweet cherry cv. Lapins leaves were treated with 0, 0.05, 0.2, or 0.5 mM SNP and inoculated with Pss (2×10^8 CFU/mL) or mock (0.01% Tween 20^{TM}). Representative images of 4-, 7-, and 10-days post-inoculation (dpi) of leaf disks from leaves sampled at 4 **(a),** 7 **(b),** and 30 **(c)** days post SNP treatment. In the mock condition, leaves with all SNP treatments are shown in the same plate (three disks per SNP treatment).

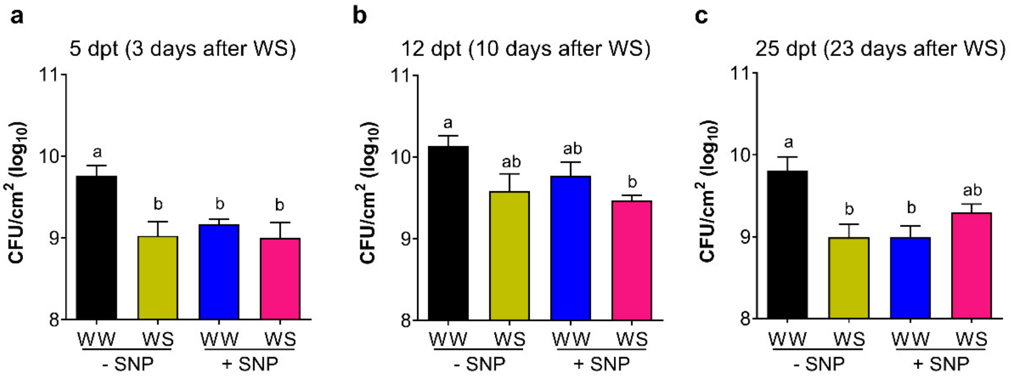

Supplementary Figure S4. Effect of exogenous nitric oxide on the viable bacterial load of Pss-infected sweet cherry leaves under water restriction. Sweet cherry cv. Lapins plants were treated with 0 mM (- SNP) or 0.5 mM (+ SNP) SNP. After two days, plants maintained their irrigation (WW), or they were exposed to a complete water shortage (WS) in two independent seasons. After that, sampled leaves were inoculated with Pss (2 x 10^8 CFU/mL). Quantification of the viable bacterial load in 10-day post-inoculation disks from leaf samples 5 **(a)** and 12 days **(b)** post-SNP treatment in the first season and 25 days post SNP treatment **(c)** in the second season. n = 9. Different letters indicate statistically significant differences in a two-way ANOVA with Tukey multiple comparisons test ($p<0.05$).

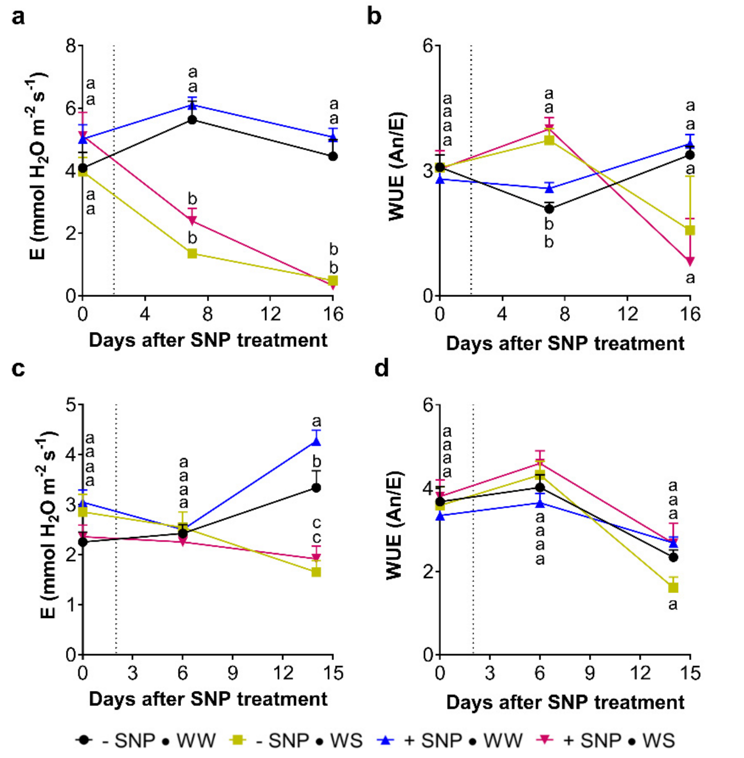

Supplementary Figure S5. Evolution of the transpiration rate and water use efficiency in sweet cherry exposed to exogenous nitric oxide under water restriction. Sweet cherry cv. Lapins plants were treated with 0 mM (- SNP) or 0.5 mM (+ SNP) of SNP. After two days, plants maintained their irrigation (WW) or were exposed to a complete water shortage (WS, denoted by a dashed vertical line) during two independent seasons. Temporal course of the transpiration rate (E; **a, c**) and the water usage efficiency (WUE; **b, d**) during water restriction assay in the first (**a, b**) and second season (**c, d**). N = 8. Different letters between means at the same time indicate statistically significant differences in a two-way ANOVA with Tukey multiple comparisons test ($p<0.05$).

Supplementary Table S1. Measured air temperature and relative humidity in the post-transplanting and the water restriction periods along two independent seasons.

	Temperature (°C)			Relative Humidity (%)		
**2021 Season**	**Max. °C**	**Min. °C**	**Mean °C**	**Max. °C**	**Min. °C**	**Mean °C**
Post-transplanting period	33.7	4.3	17.4 ± 6.85	95.2	18.5	58.3 ± 19.8
Water restriction period	35.6	9.1	19.6 ± 6.1	95.2	20.1	56.8 ± 19.3
**2022 Season**						
Post-transplanting period	34.7	3.7	15.1 ± 5.6	89.9	9.0	62.1 ± 16.6
Water restriction period	35.0	9.2	21.6 ± 6.1	85.3	18.4	52.4 ± 15.7
